# A rare case of hepatocellular carcinoma and colorectal liver metastasis

**DOI:** 10.1093/jscr/rjae701

**Published:** 2024-11-16

**Authors:** Wed K Alwabel, Saud M Aljesh, Ibrahim S Alsamaani, Ayman S Alrasheed, Nayef A Alzahrani

**Affiliations:** College of Medicine, King Saud bin Abdulaziz University for Health Sciences, Al Rimayah, Riyadh 14611, Saudi Arabia; Department of Surgery, National Guard Health Affairs, King Abdulaziz Medical City, Al Rimayah, Riyadh 14611, Saudi Arabia; Department of Surgery, National Guard Health Affairs, King Abdulaziz Medical City, Al Rimayah, Riyadh 14611, Saudi Arabia; King Faisal Specialized Hospital & Research Center, Al Mathar, Riyadh 12713, Saudi Arabia; Department of Surgery, National Guard Health Affairs, King Abdulaziz Medical City, Al Rimayah, Riyadh 14611, Saudi Arabia; Peritonectomy and Liver Unit, St George Hospital, Kogarah, NSW 2217, Australia

**Keywords:** colorectal neoplasm, hepatocellular carcinoma, neoplasm, metastasis, abdominal pain, colonic neoplasm

## Abstract

Hepatocellular carcinoma is the third leading cause of cancer deaths worldwide, with a 5-year survival rate of 20.3%, while colorectal cancer is a major cause of morbidity and mortality worldwide, being the third most common cancer in men and the second in women. In addition, multiple primary tumors, involving cancers at different sites and histologies, occur in 2.4% to 17% of cases. We report a case of a 74-year-old man with colon cancer presented at the Emergency Department with asymptomatic anemia post chemotherapy and surgical intervention two years ago. He reported experiencing paleness, dizziness, exertional dyspnea, and fatiguability for the past month. Therefore, chest computed tomography was performed to rule out pulmonary embolism; however, the image revealed an incidental finding of two hepatic lesions in segment II. After further investigations, the decision was to perform hepatic segmentectomy. Postoperative pathology revealed residual Hepatocellular carcinoma and metastatic colonic-type adenocarcinoma with mucinous differentiation.

## Introduction

Hepatocellular carcinoma (HCC) is the third most common cause of cancer-related deaths worldwide, with 5-year survival rate of 20.3% [1]. The two most important etiological contributing factors are hepatitis B and C. HCC receives most of its blood supply from branches of the hepatic artery; therefore, the characteristic radiological feature of HCC is branches from the hepatic artery. Colorectal cancer (CRC) is also a major cause of morbidity and mortality worldwide. It is the third and second most common cancer in men and women, respectively, with an overall mortality rate lower than that of HCC [2]. The liver is the most common site of CRC metastasis, arising from hematogenous spread via the portal venous system. Currently, imaging modalities, such as computed tomography (CT) and magnetic resonance imaging (MRI), are used to characterize and differentiate hepatic lesions to facilitate the best management approach. Multiple primary tumors (MPT) are defined as more than one cancer in an individual at different sites with different histologies. MPT incidence ranges between 2.4% and 17% [3]. Among multiple primaries, the incidence of colon cancer is 19.8% [3]. Colon cancer is the third most common cancer worldwide and has an excellent prognosis with a 5-year-survival rate of 80% for localized disease; however, this rate drops to 14% in metastatic disease [3]. HCC is the third most common cause of cancer death worldwide with a 5-year survival rate of 20.3%.

## Case report

**Figure 1:**
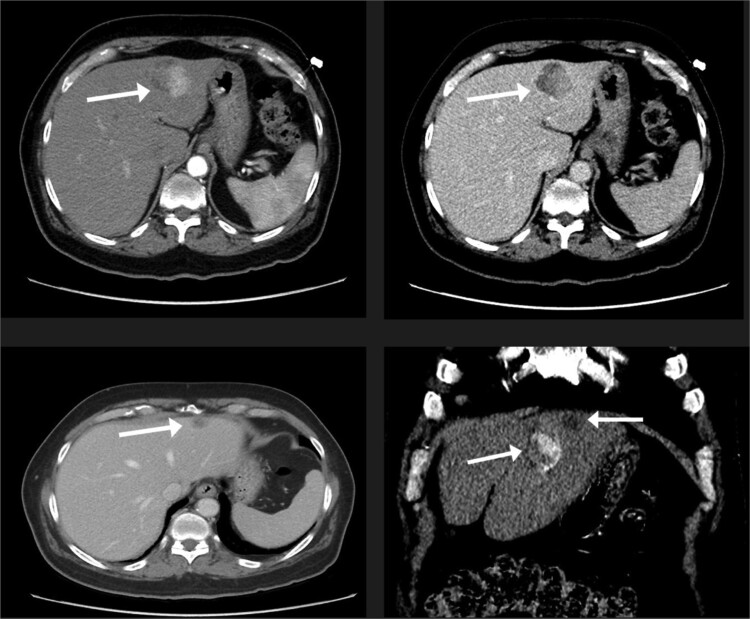
The liver lesions in segment II. The largest lesion is 3.3 × 3.4 cm and demonstrates arterial enhancement and faint washout in the delayed phase. In the portovenous phase, the smaller lesion is 1.2 × 1.1 cm.

**Figure 2:**
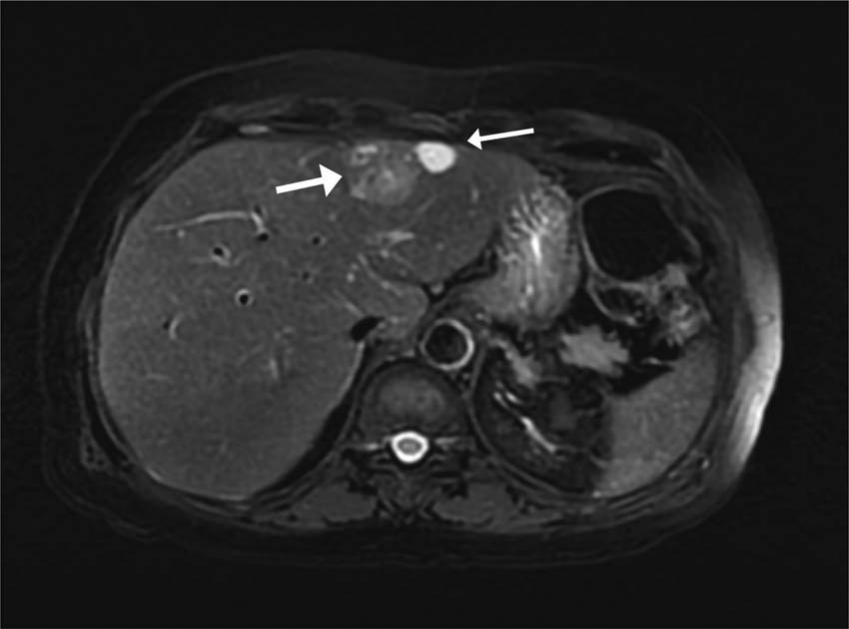
Two segment II hepatic lesions: the larger lesion is consistent with clear cell variant hepatocellular carcinoma and the second, more cranial lesion, is likely metastasis from a mucinous adenocarcinoma.

**Figure 3:**
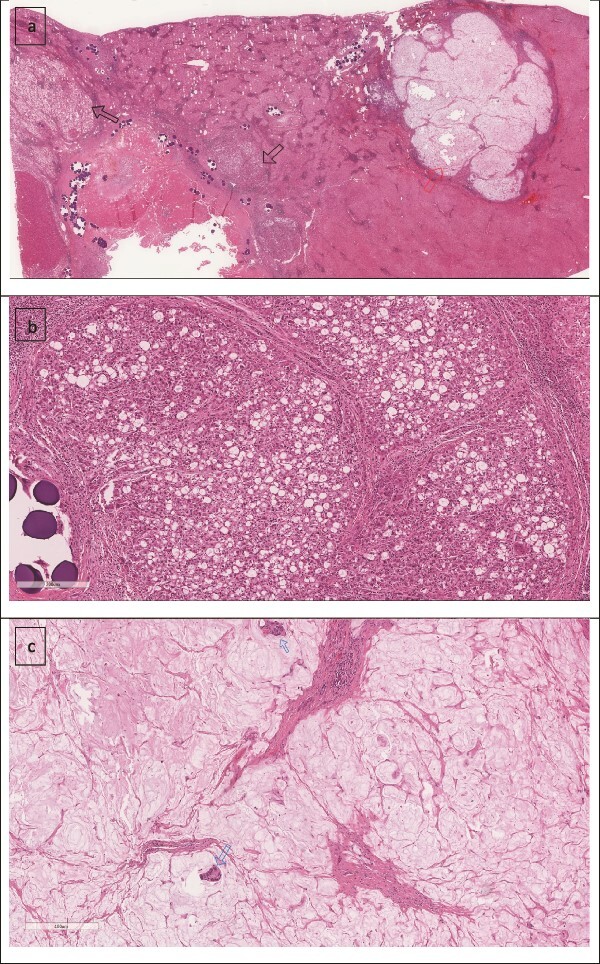
(a) Low power image showing two foci of metastatic mucinous colorectal adenocarcinoma (right arrow) and the treated hepatocellular carcinoma (left arrow). (b) Higher power image showing the hepatocellular carcinoma component. (c) Mucinous adenocarcinoma with two malignant glands.

A 74-year-old man with right-handed colon cancer presented at the Emergency Department with a symptomatic anemia following chemotherapy and surgical intervention one year prior to the hepatocellular carcinoma resection. He reported 1-month history of paleness, dizziness, and exertional dyspnea, and fatiguability. Physical examination results were unremarkable. A chest CT angiography did not reveal a pulmonary embolism; however, two hepatic lesions, measuring 3.5 cm and 1.5 cm, in segment II were noted incidentally. Therefore, further laboratory tests were performed; hepatitis C virus was detected with a liver cirrhosis Child-Pugh score indicating class A disease; however, no tumor markers were detected. An abdominal CT revealed the two hepatic lesions in segment II, the larger lesion (3.8 cm × 3.4 cm) demonstrated arterial enhancement and faint washout in the delayed phase (Fig. 1). The smaller lesion measured 1.2 cm × 1.1 cm (Fig. 1). An abdominal MRI was performed to characterize the hepatic lesions: one lesion was consistent with HCC and the second was metastatic from mucinous which was visible previously on imaging at the time of diagnosis of colon cancer (Fig. 2). Furthermore, the biopsy of the two liver lesions confirmed the diagnoses of HCC and metastatic colon cancer. Subsequently, the case was discussed within a multidisciplinary tumor board (MDTB), where the decision was made to perform chemotherapy and trans arterial chemoembolization for the HCC lesion. Upon follow-up evaluation, segment II hepatic lesion size had decreased to 1.6 × 2.4 cm (previously was 3.8 × 3.4 cm) and 1.5 × 1.3 cm (previously 1.2 × 1.1 cm). Consequently, the case was rediscussed in MDTB, and it was decided to proceed with liver resection of the two lesions. Following this, the patient underwent left lateral lobectomy, common hepatic artery dissection, porta hepatis lymph node dissection, and cholecystectomy. Postoperative pathology showed a residual 2.3-cm-sized differentiated HCC and a metastatic colonic-type adenocarcinoma with a mucinous differentiation which was 1.2 cm in size. The two lesions measured 0.7 cm apart (Fig. 3). All lymph nodes dissected from the porta hepatis, and the common hepatic artery were positive for metastatic colonic type adenocarcinoma. The patient’s postoperative course was unremarkable, and he was discharged 12 days later. Following a discussion within the MDTB, it was decided to continue adjuvant chemotherapy.

## Discussion

The treatment of patients with multiple primary tumors is challenging and, currently, there are no established treatment guidelines for such patients. Therefore, the treatment strategy for each patient should be personalized and discussed within a MDTB. In the setting of a known colorectal malignancy, a diagnosis of colorectal liver metastasis can only be made with high specificity imaging [4]. Findings are usually nonspecific and overlap with other adenocarcinoma lesions, such as intrahepatic cholangiocarcinoma and metastases from other primary tumors. Therefore, in such cases, clinicians should consider which cancer to treat first, determine appropriate therapy (medical or surgical), and develop a strategy aimed at improving patient survival.

In EORTC 40983, the survival outcomes of patients with CRC liver metastasis who received combined chemotherapy and surgery were compared with those of who underwent surgery alone for respectable lesions; the findings revealed that the progression free survival at 3 years was 8.1% for the surgery alone group and 36.2% for the peri-operative chemotherapy group [5]. Approximately 20% of patients with limited metastatic disease to the liver have the potential for surgical resection; therefore, curative treatment should be considered as resection improves the 5-year survival up to 20%–40% [5]. Hepatic resection of colorectal metastasis may be performed in a combined procedure or in two stages. Given that our patient had a second primary cancer in the liver confined to the same segment of the metastatic nodule, the MDTB had decided upon upfront surgery to control the colon primary cancer, followed by chemotherapy. Moreover, HCC management requires a thorough assessment of the patient’s general status and liver preserve. In patients without liver cirrhosis, surgical resection is the gold standard treatment option. In cases like our patient who had class A cirrhosis, surgical resection may be considered in cases of preserved liver function and in the absence of portal hypertension. Even though surgical resection remains the only curative option for liver CRC metastasis and HCC, locoregional therapy, namely transcatheter arterial chemoembolization (TACE), which is most used as a palliative therapy for unresectable lesions, has been investigated and used as a down-staging bridge to curative surgery; however, there is no significant data to support its use. In this case, the patient underwent adjuvant chemotherapy, and TACE was employed to prevent HCC progression.
